# Enhancing the quality of life for physical therapists: insights from a cross-sectional study

**DOI:** 10.3389/fpubh.2024.1286727

**Published:** 2024-03-19

**Authors:** Rahaf Eid Al Ali, Sahab A. Alrowaishd, Elaf Z. Abu Thyab, Raghad K. Almarzuqi, Manal I. Al Awaji, Monira I. Aldhahi, Zizi M. Ibrahim

**Affiliations:** ^1^Department of Rehabilitation Sciences, College of Health and Rehabilitation Sciences, Princess Nourah bint Abdulrahman University (PNU), Riyadh, Saudi Arabia; ^2^Department of Physical Therapy for Surgery, Faculty of Physical Therapy, Cairo University, Giza, Egypt

**Keywords:** functional activity, well-being, quality of life, healthcare provider, physiotherapist

## Abstract

**Background:**

Physical Therapy profession is known for its demanding physical requirements. This increases the risk of attrition and work-related accidents and disorders that affect physical therapists’ quality of life and work performance. This study aimed to evaluate the effect of physical activity level and other contributing factors on quality of life of physical therapists.

**Methods:**

A cross-sectional study was conducted among practicing physical therapists (*n* = 258). The International Physical Activity Questionnaires-Short Form was used to measure physical activity levels and the World Health Organization Quality of Life Questionnaire short form was used to measure the quality of life among physical therapists. Data was collected through a self-administered online survey using Microsoft Forms.

**Results:**

The eligible participants were 258 out of 297. The highest percentage of physical therapists had a moderate physical activity level (45.35%) and the median for overall quality of life score was 63.27(52.73–73.59). There was a significant positive correlation between physical activity and age with overall quality of life score (rs = 0.41, *p* < 0.001; rs = 0.13, *p* < 0.036) respectively and a significant negative correlation between body mass index and overall quality of life score (rs = −0.13, *p* < 0.04).

**Conclusion:**

The results obtained revealed that physical therapists mostly have moderate physical activity level and relatively good perceived quality of Life. Furthermore, our study identified significant correlations between physical activity, age, body mass index, and the overall quality of life among practicing physical therapists.

## Introduction

Quality of life (QoL) comprises an individual’s perception of satisfaction and their position in life. This includes considerations of cultural and other value systems, as well as personal goals and expectations (1, 2). Furthermore, QoL extends beyond more life satisfaction and encompasses various aspects such as personal, social, sexual, and psychological dimensions, in addition to factors like autonomy and physical health (3). Several elements influence an individual’s QoL, and one prevalent factor is physical activity (PA). The positive impact of PA on QoL extends beyond physical health, encompassing the preservation of cognitive functions, the enhancement of social relations, improvement in functionality and productivity, and boosting mood (4, 5). This positive impact is reinforced by maintaining a healthy BMI, given the bidirectional association between a high BMI and QoL, particularly in terms of mental well-being (6). Individuals with a high BMI experience general negativity toward themselves like low self-esteem and increase self-blame. Additionally, social stigma, and certain negative behaviors and attitudes toward diet further contribute to impairing social and work life and overall mental well-being (7). Unlike PA and BMI, age has a negative impact on QoL (8–11).

In the context of healthcare settings, employees face unique challenges that may have a negative impact on both physical and mental well-being. Multiple factors such as job requirements, work shifts, workload, stress levels, behavior, age, and social issues are known to play a role in this (12). Several studies in the literature reported that high work demands among healthcare workers led to less engagement in exercise and increased consumption of unhealthy food which contribute to higher rates of obesity and affect their work achievements (13, 14). Specifically, the Physical Therapy (PT) profession is known for its demanding physical requirements, including transferring and lifting patients with disabilities. This puts physical therapists (PTs) at a higher risk for attrition and work-related accidents and disorders (15). Consequently, physical therapist’s work performance can be negatively impacted leading to an elevated dependence on electrical modalities in patients’ treatment plans and seeking assistance when they handle the patients during sessions (16).

Previous studies have primarily concentrated on health-related quality of life (HR-QoL) among PTs (12, 17). This concept overlaps with QoL’s meaning. HR-QoL can be defined as those aspects of life that are affected by the presence of illness. However, there is a lack of literature assessing the QoL as experienced by PTs. Therefore, the overarching objective of the study to provide insights into the relationships between PA, various demographic and work-related factors, and the QoL of PTs in Saudi Arabia (SA). To accomplish this overarching goal, we have set out the subsequent specific aims: firstly, to characterize the pattern of PA and QoL levels among physical therapist in SA community; secondly, to analyze variations in QoL with respect to factors like PA engagement, age, gender, BMI, and work-related variables among PTs; and lastly, to explore the relationship of PA and associated factors on the QoL of PTs practicing in SA.

## Methodology

### Study design and settings

A descriptive quantitative, correlational, cross-sectional study was conducted between February and June 2022 among 258 PTs working in SA via an online English questionnaire to evaluate the level of PA and QoL among PTs working in SA. A non-probability convenience sampling method was used.

### Study population

The study population was practicing PTs in SA, from all sectors; public, private, or in both academic and clinical settings; and included all types of PT specialties.

### Inclusion and exclusion criteria

**Figure 1:**
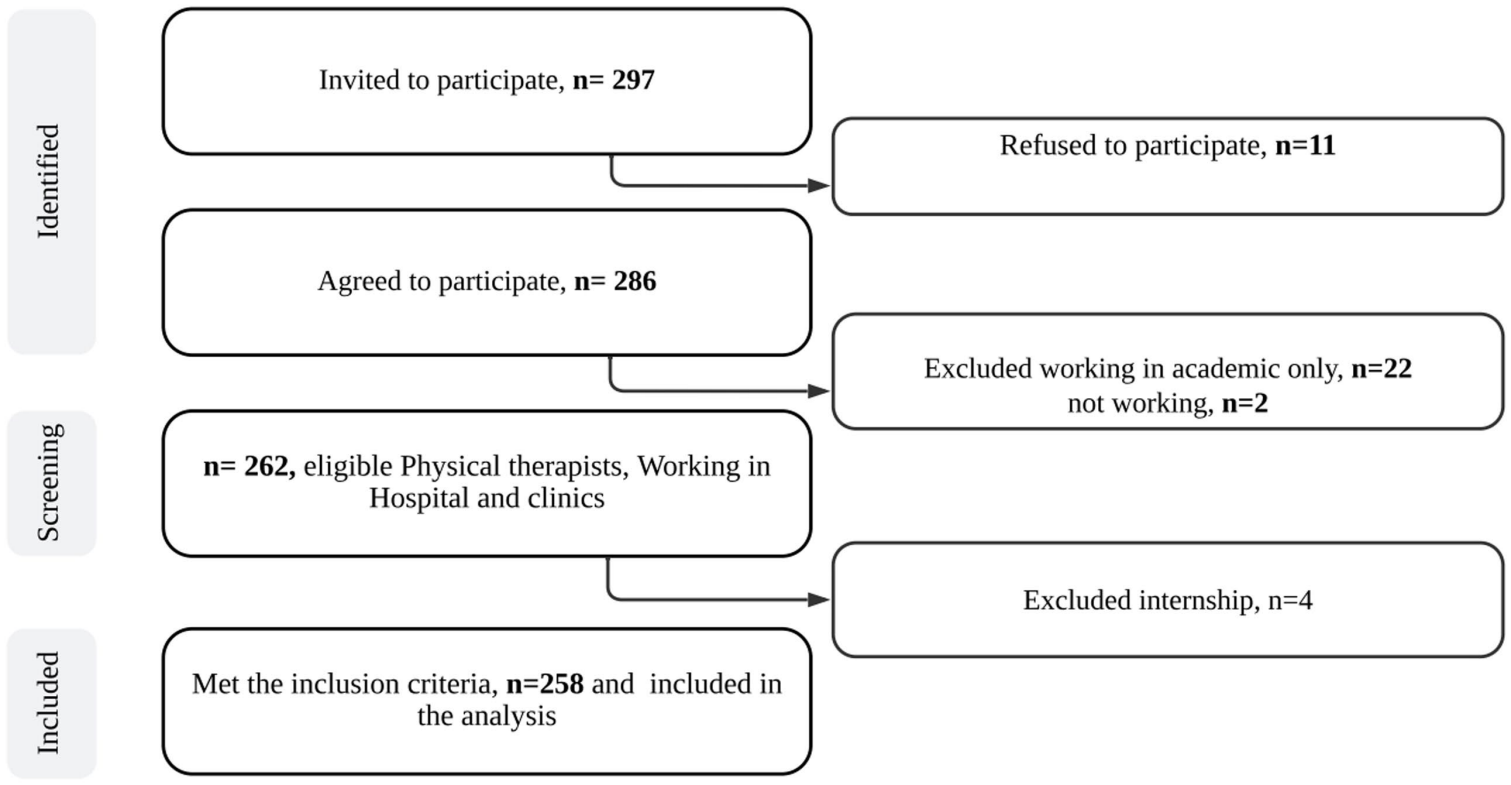
The study flowchart.

Inclusion criteria were PTs from both genders, currently working in SA, from any PT specialty, full-time or part-time in any public or private settings, or working in both academic and clinical settings. Undergraduate PTs, interns, without a license, or who are currently not working, and who are not working in SA were excluded (Figure 1).

### Sample size calculation

The sample of our study was drawn from the available pool of physiotherapists working in Saudi Arabia. Various sources were utilized to estimate the population of physiotherapists in Saudi Arabia, including data from the Saudi Commission for Health Specialties (18), and prior studies on the subject (19). In light of these considerations, the estimated number of PTs in Saudi Arabia is approximately 10,000. The anticipated representative sample size for the study was 260 therapists, determined with a confidence level of 95%, a margin of error of 0.05, and a prevalence of 80% that was calculated from a pilot study which was conducted on 30 participants and their data was not included into the study data.^1^

### Ethical consideration

This study was approved by the IRB of PNU (IRB #/22–0103) in Riyadh, Saudi Arabia, in accordance with the guidelines proposed in the Declaration of Helsinki. The permission to use the questionnaire was taken from the World Health Organization (WHO) after submitting the required forms. Prior to participating, all participants were given electronic consent on the first page of the questionnaire which outlined the aim and procedure of this study before participation. They also were notified that their participation was voluntary and assured that their data would remain confidential and anonymous, solely utilized for research purposes.

### Study procedure

The researchers created a link to the self-administrated online form (including the consent form, the demographic characteristics, (IPAQ-SF) questionnaire, and the WHOQOL-BREF questionnaire using the Microsoft Forms service). The link was shared with the target population via social media and visiting hospitals. The first page of the online survey contained the informed consent, which described the aim and objectives of the study; the respondents proceeded to fill in the form if they agreed to participate in the research study. The survey was piloted on 19 expert PTs to receive their comments regarding the clarity and understandability of the survey items and to calculate the estimated average time to fill in the questionnaire. The data from the pilot were not included in the data analysis of the main study.

### Study instruments

Data was collected using the Microsoft Forms web application. The online form consisted of four sections. The first section contained the introductory information about the research purpose and procedure, the consent form to participate in the study and the time expected to finalize the survey which was estimated to be about 10 min at maximum. The second section is the demographic characteristics which consists of 12 questions related to age, sex, height, and weight to calculate the BMI, nationality, marital status, educational qualification of PT, current professional classification, working place, working hours per day, years of experience, primary scope of practice, and the geographical location of practice. Additionally, two questions inquire about the presence of health illness and work-related musculoskeletal disorders (WR-MSDs).

The third section of the survey was the International PA Questionnaires-Short Form (IPAQ-SF) which was used to measure the level of PA using the duration and frequency of PA in the last 7 days IPAQ-SF is a valid and reliable self-reported open-ended questionnaire with an internal consistency of Cronbach Alpha = 0.647) (20). It is a free-access questionnaire developed by an international consensus group in 1998, and it is available to use without permission on the website of IPAQ (21). It consists of 7 questions related to four different types of activity, namely vigorous activities such as lifting and aerobics, moderate activities like cycling, walking as transportation or leisure and sitting at work or free time. Computation of the total score requires the summation of the PA duration (in minutes) and frequency (days) then transformed to the metabolic equivalent of task-minute per week (MET-min/week) and time spent sitting to estimate the PA level using a published formula (22). The final score (MET-min/week) will be categorized into low, moderate and high PA according to IPAQ group guidelines (23).The fourth section contains the English version of the World Health Organization Quality of Life Questionnaire short form (WHOQOL-BREF) to measure the QoL scores. It is a valid and reliable questionnaire with an internal consistency of (Cronbach Alpha = 0.896) (3, 24). The WHOQOL-BREF is a self-administered questionnaire containing 26 questions about QoL from 4 domains which are physical, psychological, social relationships, and environmental areas. The physical domain measures the discomfort facets and pain, daily life activities, sleep, rest, energy, and fatigue facets. The psychological domain includes the experience of positive and negative perceptions and what affects daily functioning, assessing the way a person thinks, capability in making decisions, self-esteem, body image, and satisfaction. The social domain evaluates the person’s relationship with social support with family and friends. The environmental domain assesses the physical safety facets, healthcare availability services, accessibility for acquiring new skills or information, enjoyment of leisure activity, and the availability of transportation. The survey uses a 5-point Likert scale where higher scores on positive facets correspond with a higher quality of life. Three questions have a reversed scoring. The cut-off point for a good QoL is 60% or above. The individual calculation for the WHOQOL-BREF score is calculated by summing of each domain individually and multiplying it by 4, then the summation of all 4 domains by the next formulas to transform it into 0–100 to agree with the WHOQOL-100 SCORES (25). Cronbach’s alpha coefficient to assess the reliability of the collected responses to the QoL questionnaire reported to have a value of 0.848.

### Statistical producers and data analysis

All statistical measures were performed using the Statistical Package for Social Science (SPSS) program (version 26.0). The data was presented in the form of numbers, percentages, median, and interquartile range. The internal consistency of the survey was assessed using Cronbach’s Alpha test. A value of *p* ≤ 0.05 was considered statistically significant. Data normality was tested using the Kolmogorov- Smirnov test. For comparing the quality of life (QoL) scores between two independent groups, the statistical test employed was the Mann–Whitney test. For comparing QoL scores among multiple independent groups, the Kruskal-Wallis test was utilized.

Spearman’s correlation was conducted to test the relationships between the QoL and PA and some related variables (age, BMI, working hours, clinical experience, and clinical hours).

## Results

### Sociodemographic characteristics of the participants

Out of 286 participants who completed the survey, 258 met the inclusion criteria and 28 were excluded because 22 were only academic, 4 were interns and 2 were not currently working. Table 1 demonstrates the characteristics of participants who completed the survey. There is an accepted variation between the number of males 46.5% and females 53.5%. The majority were Saudi 93. %, aged less than 30 years old 65.5%, single 55.8% and with a normal BMI 43.0%. Specialists represented 69% of the sample, while 27.90% were senior specialists and only 3.1% were consultants. Most of the participants 72.1% held bachelor’s degrees, 43.0% worked in governmental hospitals and only 6.6% worked in both academic and clinical fields. 61.6% had clinical experience less of than 5 years, 52.7% worked in the musculoskeletal/orthopedic, only 12.8% of the sample had medical illness, and 42.6% had WR-MSDs.

**Table 1:** Characteristics of the Participants (*N* = 258).

Variable		n	%
Gender	Male	120	46.5
	Female	138	53.5
Nationality	Saudi	240	93
	Non-Saudi	18	7
Age(years) (Minimum-maximum: 20–58)	20–30	169	65.5
	31–40	70	27.1
	>40	19	7.4
Marital statue	Single	144	55.8
	Married	104	40.3
	Divorced	10	3.9
Professional classification	Specialist	178	69
	Senior specialist	72	27.9
	Consultant	8	3.1
PT degree	Diploma	10	3.9
	Bachelor’s degree	186	72.1
	Master’s degree	45	17.4
	PHD	17	6.6
BMI (kg/m^2^)	Under weight	15	5.8
	Normal	111	43.
	Overweight	90	34.9
	Obese	42	16.3
Setting practice	Governmental hospital	111	43.
	Private hospital/clinic	106	41.1
	Academic /Governmental hospital	17	6.6
	Governmental/Private hospital/clinic	24	9.3
Number of academic working hours per day	Not academic	241	93.4
	Less than 6 h	4	1.6
	6–9 h	13	5.
Number of clinical working hours per day	Less than 6 h	49	19
	6–9 h	209	81.
Clinical experience (years)	1–5 years	159	61.6
	6–10 years	48	18.6
	11–15 years	25	9.7
	More than 15 years	26	10.1
Primary scope of practice	General	11	4.3
	Cardiology	5	1.9
	Musculoskeletal / orthopedic	136	52.7
	Neurologic	41	15.9
	Pediatric	33	12.8
	Sport	25	9.7
	Women’s health	7	2.7
Region	Central region	149	57.8
	Western region	51	19.8
	Eastern region	24	9.3
	Southern region	20	7.8
	Northern region	14	5.4
Medical illness	Yes	33	12.8
	No	225	87.2
work-related musculoskeletal disorder	Yes	110	42.6
	No	148	57.4

### PA and overall QoL score among participants

The total scores of different PA of studied participants are presented in Table 2. The median scores of walking, moderate, vigorous activities and sitting were (330, 80, 0.00 min/week, 4 h) with interquartile range of (99–693, 0.00–480, 0.00–240, 3–7) respectively. Regarding the distribution of PA levels, (45.35%) have a moderate level of PA, followed by a low level (41.86%), then a high level (12.79%).

**Table 2:** Total scores of different physical activity among participants (*N* = 258).

Physical activity level (MET-minutes/week)	Median (interquartile ranges)
Walking	330 (99–693)
Moderate activity	80 (0.00–480)
Vigorous activity	0.00 (0.00–240)
Total physical activity	693 (282.525–1481.75)
Sitting (hours)	4 (3–7)
**Distribution of physical activity levels in the studied sample (n, %)**	
High (MET > 2,999) OR > 1,500 vigorous	33 (12.79%)
Moderate (MET 600–2,999)	117 (45.35%)
Low (MET < 600)	108 (41.86%)

Keys: Walking MET-minutes/week = 3.3 * walking minutes * walking days. Moderate MET-minutes/week = 4.0 * moderate-intensity activity minutes * moderate days. Vigorous MET-minutes/week = 8.0 * vigorous-intensity activity minutes * vigorous-intensity days. Total physical activity MET-minutes/week = sum of Walking + Moderate + Vigorous MET-minutes/week scores.

Table 3 shows the mean row scores, medians, and interquartile range of the transformed scores of each item on WHOQOL-BREF. The physical health and social relationships domains have the highest median (67.86, 66.67) with interquartile ranges of (53.57–78.57, 50–75) respectively. While the psychological health and environment domains have the lowest median (62.5, 62) with interquartile ranges of (50–75, 50–71.88) respectively. In addition, The Overall QoL median score was 63.27 with an interquartile range (52.73–73.59).

**Table 3:** Description of WHOQOL-BREF domains among participants (*N* = 258).

WHOQOL-BREF items/domains	Mean row score	Transformed median (interquartile ranges)
Q1 Overall QOL	3.51	62.5 (50–75)
Q2 General health	4.12	75 (50–100)
Domain 1: Physical Health	3.62	67.86 (53.57–78.57)
Domain 2: Psychological Health	3.43	62.5 (50–75)
Domain 3: Social Relationships	3.5	66.67 (50–75)
Domain 4: Environment	3.46	62 (50–71.88)
Overall QoL score		63.27 (52.73–73.59)

### Comparison of QoL across the participants’ characteristics

Table 4 reveals the comparison of the overall QoL score across various participants’ characteristics. It was found that Age, BMI, and PT degree categories showed significant differences in the overall quality scores (*p* = 0.02, 0.02, 0.008, respectively). Moreover, significant differences in QoL scores were observed between different levels of PA levels (*p* < 0.001). Conversely, no significant differences were identified in the overall QoL scores within gender, marital status, and the presence of medical illness categories (*p* = 0.45, 0.16, 0.5, respectively).

**Table 4:** Comparison of QoL between the participants’ characteristics.

Variables		Overall QOL score	Value of *p*
Gender	Male	63.67(56.6–73.2)	0.45
	female	63.04(54.6–73.6)	
Age (years)	20–30	62.9(56.8–73.8)	0.02*
	31–40	64.1(57.7–72.9)	
	>40	71.61(57.2–78.4)	
Marital status	Single	63.55(57.2–73.8)	0.16
	Married	63.12(45.9–73.9)	
	Divorced	66.06(56.6–76.6)	
PT degree	Diploma	62.84(60.8–77.6)	0.008**
	Bachelor	69.78(60.4–78.4)	
	Master	73.30(65.1–78.74)	
	PhD	78.57(71.74–88.1)	
BMI (kg/m^2^)	Under weight	58.97(53.60–74.6)	0.02*
	Normal	63.99(58.8–70.9)	
	Over weight	64.04(56.3–75)	
	Obese	61.4(56.3–69.4)	
Medical illness	Yes	61.8(52.5–73.1)	0.53
	No	63.1(53.3–75.1)	
Physical activity level	low	53.92(47.4–63.06)	<0.001***
	Moderate	69.79(60.9–67.4)	
	High	70.72(58.9–77.4)	

Statistical tests were Mann Whitney for comparing of two groups and Kruskal Wallis for comparing multiple group: *p ≤ 0.05; **p ≤ 0.01; ***p ≤ 0.001. Data presented as Median (interquartile ranges).

Table 5 illustrates the comparison of overall Quality of Life (QoL) scores among work-related variables of the participants. No significant differences were observed in overall QoL scores between various professional classifications and working hours per day categories (*p* = 0.06, 0.49, respectively). However, there were significant differences in overall QoL related to clinical experience years (*p* = 0.04). Regarding Work-Related Musculoskeletal Disorders (WR-MSDs), no significant differences were identified in overall QoL scores (*p* = 0.11).

**Table 5:** Comparison of QoL between the participants’ work-related factors.

Variables		Overall QOL score	Value of *p*
Professional classification	Specialist	63.1(52.8–72.4)	0.06
	Senior specialist	63.6(51.6–74.6)	
	Consultant	74.7(64.9–87.5)	
Working hours per day	<6 h	61.9(49.9–78.7)	0.49
	6–9 h	63.1(52.9–80.6)	
Clinical experience	1–5 years	63.1(53.5–73.1)	0.04*
	6–10 years	59.5(50.1–69.5)	
	11–15 years	63.2(51.9–71.7)	
	>15 years	71.6(59.7–83.3)	
Work-related musculoskeletal disorder	Yes	62.5(50.4–73.5)	0.11
	No	63.5(53.5–73.9)	

Statistical tests were Mann Whitney for comparing of two groups and Kruskal Wallis for comparing multiple group: *p ≤ 0.05; Data presented as Median (interquartile range).

### The correlation between age, BMI, work related variables, physical activity and QoL value

The correlation between the overall QoL score and numerical variables is presented in Table 6. There were significant low positive correlations between the age and overall QoL score (rs = 0.13, *p* = 0.036). While PA exhibited a significant association and moderate positive correlations with the overall QoL score (rs = 0.412, *p* < 0.001). In contrast, BMI has a low negative correlation with the overall QoL score (rs = −0.13, *p* = 0.04).

**Table 6:** Correlation between age, BMI, work related variables, physical activity and QoL score in the studied sample.

Variables	Overall QoL score	
	rs	Value of *p*
Age(years)	0.13*	0.036
BMI (kg/m^2^)	−0.13*	0.04
Academic working hours per day	−0.01	0.90
Clinical working hours per day	0.09	0.15
Clinical experience(years)	0.11	0.09
Physical activity (METs)	0.412* *	<0.001

*Correlation is significant at the 0.05 level (*p* ≤ 0.05), **Correlation is significant at the 0.01 level (*p* ≤ 0.01), Spearman correlation.

## Discussion

Quality of life is often impacted by various factors within the purview of healthcare providers, and it is crucial to evaluate these elements in order to enhance the quality of patient care (22). This study was conducted to explore the relationship of PA, age, BMI, and work-related factors, on the QoL of PTs. This could help to identify areas of weakness in a person’s life, ultimately leading to QoL improvement (26). Overall, the present study shows that PTs are engaged in moderate PA level, and relatively good perceived QoL and satisfaction with health (25). Thus, the QoL was significantly different among PT based on the duration of work experience and education, PA level, BMI and age. Among these factors, the QoL was correlated positively with age and PA and negatively correlate with BMI. Moreover, the results showed that PA, age, and BMI appeared to explain the variance in QoL among PTs.

Finding of this study showed that PTs had a moderate PA level and their level of PA reported to be associated with QoL. The current study’s outcomes concerning physical activity (PA) levels among PTs and their subsequent influence on quality of life align with earlier research (1, 27). These studies have demonstrated a significant positive correlation between PA and quality of life. Taken together, these findings emphasize the essential role of participating in physical activity in preserving overall health and well-being (28). While the lack of PA can negatively impact one’s physical, spiritual, and mental health and wellness in contrast, engaging in regular PA not only has a positive influence on physical health, but also enhances work performance and overall OoL (4).This active engagement in PA by PTs can be explained by their exposure to physical education during their training and their firsthand understanding of the positive impact that PA has on one’s health and overall well-being.

One of the most notable findings from the present study is the positive correlation between age and overall QoL. These finding aligned with previous study which showed that Positive perception of aging is a key predictor of QoL (29). However, this contrasts with other studies involving diverse community groups that have reported a negative correlation between age and QoL (8, 9). These discrepancies in findings may stem from variations in study populations, methodologies, or other unexplored factors. Further research is necessary to investigate and reconcile these disparities in the relationship between age and QoL. It is noteworthy that a significant portion of the respondents in this study did not report any medical illnesses or comorbidities and exhibited high scores in both physical and social domains of QoL, as well as in overall QoL. These results provide substantial support for the well-being of physical therapists in general. Additionally, most of the respondents consisted mainly of young and physically active PTs which most likely contributed to their favorable health status. Future studies are recommended to explore the broader spectrum of age-related differences in QoL to gain a more comprehensive understanding of these variations.

Our results revealed no significant difference in QoL score between females and males. Finding of lack of gender disparities in QoL was not aligned with the pervious study in which significant differences in the levels of quality of life was establish between men and women (30). These differences were partly explained by sociodemographic, clinical, and behavioral factors. It is possible that these variations are a result of a socio-historical process influenced by gender-related factors that have shaped the roles and expectations associated with work. These contradicting findings could be attributed to unmeasured variables or factors not included in our study might contribute to the observed differences in quality of life between men and women. Additionally, cultural, or societal factors that vary across different populations may also play a role in shaping these disparities. Further investigation is warranted to explore these potential explanations.

Thus, the BMI negatively correlated with the QoL among PT. Numerous studies revealed that a high BMI has an adverse impact on QoL compared to adults with a normal BMI (31, 32). A meta-analysis found that high BMI is significantly correlated with lower scores in QoL, particularly in the physical and psychological health domain (32). The result of the current study demonstrates the same negative correlation between overall QoL score and BMI, predominantly in the psychological domain. This can be explained by the complex bidirectional relationship between BMI and psychological well-being (33). Finding also confirm the findings in the previous population-based study that investigate the association between obesity and quality of life (34). The inverse independent association between increasing weight status and decreasing QoL has confirmed that long-term conditions play a mediating role in the reduction of QoL in individuals with obesity (35). These results implying that individuals with obesity who are considered “healthy” may be transitioning toward an unhealthy future.

Finally, it is important to note that an employee’s positive impact on quality of life (QoL) is not solely confined to their work life (36). This aligns with the spillover hypothesis, which suggests that the circumstances and experiences encountered in work can spill over to other domains of life and vice versa (37). Moreover, the detrimental effects of high BMI and work-related musculoskeletal disorders (W-RMSDs) can negatively influence work performance, leading to increased absenteeism and work-related issues (38). In a qualitative study conducted by da Silva Pinheiro et al. in 2020, emphasized the need for strategies to enhance the quality of life and work for healthcare professionals, highlighting the significance of mental and physical well-being, as well as the provision of necessary resources for their job (39).

It is important to highlight the strengths and limitations of this study. It is considered the first study to investigate the influencing factors on the QoL of PTs in SA with an acceptable response rate. However, some limitations of the current study could be addressed in the future. The majority of the respondents were young and from the central region with less than 5 years of experience (61.63%), which limits the ability to generalize the data to the whole population. The absence of the profession’s age spectrum in the study does introduce a limitation, as it narrows the scope of applicability regarding generational practice variations. This could be attribute to many factors such as the younger physiotherapists may be more accessible and inclined to participate in studies due to interests in academic advancements or professional development. The higher response rate from younger physiotherapists in studies using electronic surveys could be attributed to their greater comfort and proficiency with digital platforms and devices. It is worth considering that the experiences and perspectives of established professionals could differ substantially due to longer career exposure and potential variations in educational backgrounds. To address this gap, we suggest a follow-up study including a broader age range to compare and contrast the results. This would provide a more holistic view of the physiotherapy landscape and offer insights into the evolution of practice patterns over time. Additionally, the study relied on self-reported data, which may be influenced by social desirability and recall bias, as the IPAQ is based on the past 7 days and the WHOQOL-BREF on the past month. Furthermore, the study fails to include the economic background of the participants, which could potentially influence their quality of life.

## Conclusion

Our cross-sectional study sheds light on the significant relationship between physical activity, age, body mass index, and the overall quality of life among physical therapists. The findings highlight the importance of maintaining a balanced lifestyle especially as grow older, in order to improve their overall quality of life. The significance of our study lies in its contribution to the broader comprehension of the diverse factors that impact the well-being of healthcare professionals, with a specific focus on physical therapists. This research carries the potential to exert a substantial influence on healthcare policies and practices. Its objective is to lay the groundwork for a more supportive and sustainable environment for physical therapists, thereby contributing to enhance overall well-being and performance of healthcare professionals operating in demanding work settings.

### Recommendations

These findings are considered as a foundation for more broadly representative future research that includes elder participants than the current study with an equal number of participants. Further studies are recommended to evaluate other detailed factors that influence QoL including professional, psychosocial and environmental factors. Additionally, a qualitative study is also recommended to shed some light on the reason for the presence of low PA levels, high BMI and low QoL among PTs.

## Data availability statement

The raw data supporting the conclusions of this article will be made available by the authors, on reasonable request.

## Ethics statement

The studies involving humans were approved by Institutional Review Board (IRB), Princess Nourah bint Abdul Rahman University (PNU). The studies were conducted in accordance with the local legislation and institutional requirements. The participants provided their written informed consent to participate in this study.

## Author contributions

REA: Conceptualization, Investigation, Methodology, Writing – original draft. SA: Conceptualization, Investigation, Methodology, Writing – original draft. ET: Conceptualization, Investigation, Methodology, Writing – original draft. RKA: Conceptualization, Investigation, Methodology, Writing – original draft. MAw: Conceptualization, Investigation, Methodology, Writing – original draft. MAl: Funding acquisition, Supervision, Validation, Writing – review & editing. ZMI: Conceptualization, Data curation, Funding acquisition, Methodology, Project administration, Software, Supervision, Writing – original draft, Writing – review & editing.

## Footnotes

^1^ www.raosoft.com/samplesize.html
